# Association of Multimodal Collateral Profiles with Outcomes After Large Core Thrombectomy

**DOI:** 10.3390/jcm15218730

**Published:** 2026-11-09

**Authors:** Rahul R. Karamchandani, Liang Wang, Gary Defilipp, Jonathan D. Clemente, Milan T. Quinn, Jeremy B. Rhoten, Dale Strong, Andrew W. Asimos

**Affiliations:** 1 Department of Neurology, Wake Forest University School of Medicine, Advocate Health, Charlotte, NC 28203, USA; milan.quinn@advocatehealth.org; 2 Clinical Quality Analytics, Advocate Health, Charlotte, NC 28203, USA; liang.wang1@advocatehealth.org (L.W.); edwin.strong@advocatehealth.org (D.S.); 3 Department of Radiology, Charlotte Radiology, Wake Forest University School of Medicine, Advocate Health, Charlotte, NC 28203, USA; gary.defilipp@charlotteradiology.com (G.D.); jonathan.clemente@advocatehealth.org (J.D.C.); 4 Department of Neurology, Carolinas Medical Center, Advocate Health, Charlotte, NC 28203, USA; jeremy.rhoten@advocatehealth.org; 5 Department of Emergency Medicine, Wake Forest University School of Medicine, Advocate Health, Charlotte, NC 28203, USA; andrew.asimos@wfusm.edu

**Keywords:** large core, collateral circulation, endovascular thrombectomy

## Abstract

**Background/Objectives**: Cerebral collateral circulation markers in large core thrombectomy and their impact on functional outcomes have not been extensively reported. **Methods**: We retrospectively analyzed health system medical records of large core (Alberta Stroke Program Early Computed Tomography Score <6 and/or computed tomography perfusion [CTP] cerebral blood flow <30% volume ≥50 milliliters [mL]) thrombectomy patients over a 19-month period. A good collateral profile (CCC+) was defined as the combination of computed tomography angiography Tan arterial score ≥2 and CTP cerebral blood volume index (CBVI) ≥0.8. A poor profile (CCC−) was defined as Tan <2 and CBVI <0.8. All others were categorized as having a mixed profile (CCC±). Multivariable Firth’s penalized logistic regression was used to identify factors independently associated with the primary outcome, 90-day modified Rankin Scale (mRS) 0–3. **Results**: 88 patients with median (interquartile range, IQR) age 65.5 (56.5–77.5) years and core volume 78 (55–116) mL were included. Forty-seven patients (53.4%) had poor collateral profiles, 31 (35.2%) were classified as CCC±, and 10 (11.4%) as CCC+. In the multivariable Firth’s penalized logistic regression model, CCC+ patients had higher odds of achieving a 90-day mRS of 0–3 compared with CCC− patients, though the difference was not statistically significant (odds ratio [OR] 1.117, 95% confidence interval [CI] 0.235–5.311, *p* = 0.889). No significant difference was observed between CCC± and CCC− patients (OR 0.869, 95% CI 0.293–2.577, *p* = 0.800). Glucose was the only factor independently associated with 90-day outcomes (OR 0.742, 95% CI 0.602–0.915, *p* = 0.005). **Conclusions**: No independent association was detected between favorable or mixed cerebral collateral circulation and 90-day mRS 0–3 after large core thrombectomy, though the study was underpowered for the CCC+ comparison. Presenting glucose may inform the likelihood of achieving independent ambulatory status at 90 days.

## 1. Introduction

Cerebral collateral circulation is associated with functional outcomes after large vessel occlusion (LVO) stroke [1,2]. Arterial measures on single- and multi-phase computed tomography angiography (CTA) [3,4], venous outflow parameters [5,6], and tissue-level collaterals quantified with computed tomography perfusion (CTP) [7,8,9,10,11,12], have all been linked with improved functional outcomes after occlusion of an intracranial artery. In addition to their individual importance, a combination of favorable anatomy encompassing the entire “collateral cascade” has been shown to better predict outcomes than mixed or poor collateral status measured at the arterial, venous, and tissue levels in patients undergoing endovascular thrombectomy (EVT) [13].

Recent randomized clinical trials have demonstrated the efficacy of EVT compared with medical management in patients presenting with large core infarctions [14], though the characterization of collateral profiles in patients with a large established stroke and their association with neurological outcomes has not been extensively studied. One analysis of patients presenting with ultra-large core infarctions ≥ 100 milliliters (mL) showed the cerebral blood volume index (CBVI), a measure of tissue-level collaterals on CTP, was independently associated with improved outcomes at hospital discharge [8]. Others have shown associations with improved outcomes and CTA- or digital subtraction angiography (DSA)-based collateral scores [15,16]. Despite the connection between favorable tissue-level collaterals and improved outcomes in some large core studies [8,15,16], and a sub-analysis of SELECT2 suggesting an interaction with collateral circulation and thrombectomy in the 0–6 h time-window [17], collateral status did not modify the treatment effect of EVT in a sub-analysis of the large core TENSION trial [18] or in a meta-analysis of 6 large core EVT studies that included SELECT2 [19].

Given the relative paucity of data on collateral circulation among patients with large core infarctions, and the observed poor functional outcomes after large core thrombectomy compared to those with small and medium cores [19,20,21], we assessed whether collateral patterns of large core thrombectomy patients were associated with functional outcomes.

## 2. Materials and Methods

### 2.1. Study Objectives and Regulatory Approval

This was a retrospective cohort study of adult acute stroke patients treated at two health system thrombectomy centers for a large core infarction from 1 January 2024 to 31 July 2025. The objectives of the study were to describe arterial and tissue-level collateral profiles of large core thrombectomy patients and determine whether a combination of favorable collateral scores were associated with improved odds of achieving independent ambulatory status at 90 days.

Institutional review board (IRB) approval was obtained from the health system (IRB00137663). While data were collected as part of the health system code stroke registry through 31 July 2025, analysis was performed after IRB approval was obtained on 12 September 2025. Patient/legally authorized representative consent was not required given the retrospective study design and absence of identifiable data.

### 2.2. Inclusion Criteria and Variables

Patients aged ≥ 18 years with an internal carotid artery (ICA) or middle cerebral artery (MCA) occlusion were included. A large core infarction was defined as computed tomography (CT) Alberta Stroke Program Early CT Score (ASPECTS) < 6 and/or CTP core infarction (cerebral blood flow [CBF] < 30% compared to the contralateral hemisphere) ≥ 50 mL. Large core infarction patients were generally considered for EVT if their pre-morbid, modified Rankin Scale (mRS) score was 0–1 and they were between 18 and 85 years old, though other patients may have been treated per the discretion of the clinical team. The modified treatment in cerebral ischemia (mTICI) score [22] was obtained by the treating neuro-interventionalist after EVT, and revascularization was defined as mTICI 2b-3. Functional outcomes as measured by mRS were obtained by trained raters at 90 ± 15 days by either telephone or electronic medical record review. The primary outcome was 90-day mRS of 0–3.

Data were obtained from a prospectively collected code stroke registry and included patient demographics, comorbidities, initial National Institutes of Health Stroke Scale (NIHSS) score, presenting glucose, as well as imaging parameters and time metrics. CTP was performed prior to thrombectomy with iSchemaView RAPID AI (Version 6.6.4, Menlo Park, CA, USA). The Tan arterial score (0–3) was calculated for each patient on pre-thrombectomy CTA by consensus between a board-certified neuroradiologist (G.D.) and vascular neurologist (R.R.K.), with higher scores indicating more robust collateral circulation [23]. Tissue-level collaterals were assessed with pre-thrombectomy CBVI on CTP. A poor cerebral collateral cascade score (CCC−) was defined as the combination of poor arterial collaterals (Tan < 2) and CBVI < 0.8; CCC+ was defined as good arterial and tissue collaterals (Tan ≥ 2 and CBVI ≥ 0.8); and the remainder of collateral profiles were categorized as CCC±. The Tan and CBVI thresholds were selected based on their original descriptions [23] and association with clinical outcomes in previously reported thrombectomy literature [10].

### 2.3. Statistical Analysis

Baseline characteristics for all included subjects, including clinical and radiographic measures, were reported using medians with interquartile range (IQR), means (±standard deviation [SD]), or counts with percentages, where appropriate. Baseline characteristics were also compared among the collateral scoring groups (CCC−, CCC+, and CCC±) using ANOVA, Kruskal–Wallis, and Fisher’s exact test, as appropriate.

We used multivariable Firth’s penalized logistic regression to identify factors independently associated with 90-day mRS of 0–3, adjusting for collateral status, age, NIHSS score, and admission glucose. The variable selection justification and model evaluation can be found in the Supplementary Materials. Because the proportion of missing data was ≤10% for variables with missing values, median imputation was used. Details of the method used to handle missing variables can be found in the Supplementary Materials. Results were reported as odds ratios (ORs) with 95% confidence intervals (CIs). A 2-tailed *p*-value of < 0.05 was considered statistically significant.

We evaluated the correlation between Tan score and CBVI using Spearman’s rank correlation coefficient. All analyses were performed using Stata (Version 19.5, StataCorp, College Station, TX, USA).

## 3. Results

**Figure 1:**
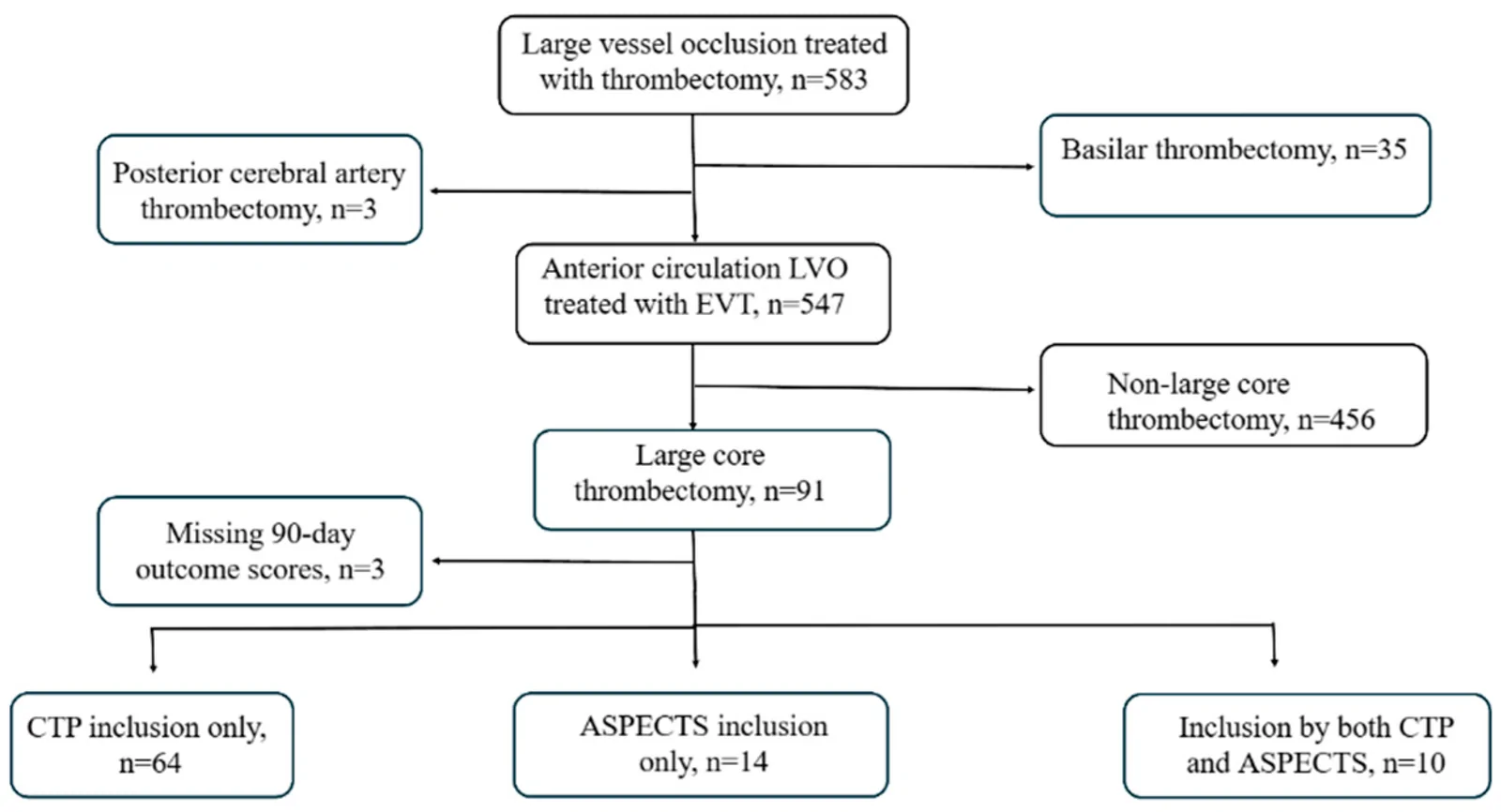
Patient flow diagram for study inclusion. n, number; LVO, large vessel occlusion; EVT, endovascular thrombectomy; CTP, computed tomography perfusion; ASPECTS, Alberta Stroke Program Early Computed Tomography Score.

During the study period, 583 patients were treated with thrombectomy, of whom 91 had an ICA and/or MCA occlusion and a large core infarction. Eighty-eight had 90-day outcome data available (Figure 1). As shown in Table 1, median (IQR) age was 65.5 (56.5–77.5) years; median NIHSS was 19 (15–23); median ASPECTS was 8 (5–10); median ischemic core volume was 79.5 (57–117) mL; and median TLKW to skin puncture was 325 (167–738) min.

Patient stratification by collateral status is shown in Table 2. Forty-seven patients (53.4%) had poor collateral profiles (CCC−), 31 (35.2%) were classified as CCC±, and 10 (11.4%) as CCC+. Groups were balanced with respect to age, initial NIHSS score, glucose, and TLKW to skin puncture, though differences were noted among smoking history, site of occlusion, and CT ASPECTS.

In the multivariable Firth’s logistic regression model, CCC+ patients had higher odds of achieving a 90-day mRS of 0–3 compared with CCC− patients, though the difference was not statistically significant (OR 1.117, 95% CI 0.235–5.331, *p* = 0.889; Table 3). No significant difference was observed between CCC± and CCC− patients (OR 0.869, 95% CI 0.293–2.577, *p* = 0.800). Glucose was the only factor independently associated with 90-day outcomes (OR 0.742, 95% CI 0.602–0.915, *p* = 0.005). CBVI was moderately correlated with the Tan score (Spearman’s ρ = 0.42, *p* <0.01; n = 91, including the 3 patients with missing 90-day mRS scores).

## 4. Discussion

In this retrospective study of large core thrombectomy patients, most subjects had poor collateral circulation profiles. No independent association was detected between favorable or mixed cerebral collateral circulation and 90-day mRS 0–3, though the study was underpowered for the CCC+ comparison. Presenting glucose was the only factor associated with 90-day outcomes.

Given the relatively higher rates of poor outcomes after thrombectomy in the large core population compared with non-large core counterparts, prognostic markers are a useful clinical tool. As recently reported, core size itself is considered “prognostic” in that it informs a patient’s clinical outcome, while penumbra is “predictive” as it relates to therapeutic response to a specific treatment [24]. As all patients in our study were treated with thrombectomy, and the treatment effect of EVT was thus not under scrutiny, we sought to determine whether a favorable collateral circulation profile was prognostic. Though this was not demonstrated, potentially related to lack of power for the CCC+ comparison, additional studies may build on our findings regarding the value of collateral circulation markers in the setting of a large ischemic core.

Recently published analyses suggest better outcomes in revascularized large core patients with improved collaterals [15,16]. Gallardo A et al. reported on an exclusively early-window (0–6 h) population with MRI core > 50 mL or CTP core >70–150 mL, in a relatively smaller sample size (n = 54) than our study. Lai H et al., meanwhile, reported on an exclusively Chinese population with ASPECTS < 6 and examined both CTA- and DSA-based collaterals. These important differences in patient populations, time windows, imaging inclusion, and sample size may explain our discrepant findings, which also may be accounted for by the lack of power for the CCC+ comparison. Additional confirmatory or conflicting findings are informative as they can guide future studies of collateral circulation markers and other potential prognosticators in large core thrombectomy.

Although collateral circulation has previously been reported to impact a patient’s clinical trajectory, “buying time” for revascularization [13], profiles in large core patients are not well defined. When a patient with a large core infarction presents, the established ischemic injury may be the result of poor baseline collateral circulation, versus that which has failed. Others have reported that the degree of collateralization and amount of salvageable tissue despite a large core infarction is variable [25]. A meta-analysis of 6 large core thrombectomy trials showed that over 60% of subjects had mismatch volumes of at least 15 mL and mismatch ratios of ≥ 1.8, implying a “large core, large penumbra” population [19]. Similarly, our patients had median ischemic core and Tmax > 6 s volumes of 78 mL and 185 mL, respectively. As most patients in our cohort had large volumes of salvageable tissue, and very few had good collateralization judged by the combination of single-phase CTA and CTP, large core patients with poor or mixed collateral profiles may still present with significant penumbra. Future trials are required to further explore the relationship between collateral circulation and infarct growth rate as it pertains to penumbral volumes and large core patient outcomes.

Moreover, the discordance between core size measured by ASPECTS and CTP is illustrated in our study. Most patients were included using only CTP criteria, while 24 were included using only ASPECTS or both CTP and ASPECTS. As such, the CCC− group paradoxically had a higher median ASPECTS (9) compared with the CCC+ group (5.5), despite larger CTP core volumes. The limitations of CTP core volume assessments have previously been described and may be a factor in the demonstrated efficacy of large core thrombectomy versus medical management (the “large core paradox”) [25]. Additional studies to better quantify core size, such as those utilizing net-water uptake [26], are required to better understand the true volume of infarcted and at-risk tissue.

In an unselected population of LVO patients, most of whom were treated with thrombectomy, a combination of favorable collateral circulation measures was associated with better functional outcomes compared with those that had either moderate or poor collaterals [13]. This profile was defined as arterial filling on CTA of ≥ 50%, cortical vein opacification score (COVES) 3–6 [5], and HIR ≤ 0.4 [13]. While alternate measures of collaterals may have yielded different results, we opted to study the CBVI rather than HIR, given the previously reported prognostic significance of CBVI in patients with ultra-large core infarctions [8]. In our study, HIR was not associated with 90-day mRS of 0–3. COVES has proven value [5], though its technically challenging calculation does not make it ideal as a prognostic marker in the acute stroke setting. Manually calculated measures, including Tan, COVES, and prolonged venous transit on CTP [6], have the potential for automated calculation through artificial intelligence (AI), similar to existing tissue-level collateral markers on CTP [10]. Dedicated studies of AI-derived collateral measures will ultimately be necessary to determine their clinical utility.

Previously, we have reported that in patients being transferred for thrombectomy, higher CBVI independently predicts 90-day functional independence, as does CBVI > 0.7 in patients who achieve recanalization [10]. However, CBVI > 0.7 was not independently associated with mRS 0–2 in the overall study cohort, nor was the combination of favorable CBVI and HIR [10]. Separately, in patients with ultra-large core infarctions (CTP core ≥ 100 mL), we have reported that higher CBVI and lower glucose were both independently associated with mRS 0–3 at discharge [8]. The present study demonstrates that the combination of CBVI dichotomized at ≥0.8 and Tan score ≥ 2 is not associated with 90-day mRS 0–3, though admission glucose is. Notable differences between the previous and current cohorts include the exclusive inclusion of patients transferred for thrombectomy and investigation of the combination of CBVI and HIR, rather than CBVI and Tan [10]; and the study of those with ultra-large core volumes, irrespective of treatment with EVT or medical management [8]. Despite these differences, our present findings reinforce these prior results, though the dichotomization at ≥0.8 may have attenuated the predictive ability of CBVI in the cohort.

Glucose, meanwhile, has been linked to poor neurological outcomes in general stroke populations [27] and in those undergoing EVT [28,29,30]. This association was reproduced in our selected population of large core EVT patients. Penumbra, specifically, is vulnerable to hyperglycemia as the glycolysis that results from reduced blood flow produces acidosis, making the tissue more likely to progress to infarction [31]. Elevated glucose is likely related to a stress response and is observed after ischemic stroke in both diabetic and non-diabetic patients [31]. It should be noted that the proportion of patients with diabetes in our study was not significantly different among the 3 collateral scoring groups, despite the association of hyperglycemia with worse outcomes. While treatment of hyperglycemia in the acute stroke setting has not been shown to improve neurological outcomes [32], the presenting glucose level has prognostic value, particularly in a population of stroke patients that have a high proportion of poor neurological outcomes.

Our study is limited by the retrospective design, with multiple potential biases, and inclusion of data from a single health system. Unmeasured confounders may have impacted our findings, such as potential neurological worsening post-thrombectomy related to clinical factors (i.e., hypotension) or social determinants of health. Although standardized guidelines were used for large core thrombectomy consideration, treatment decisions were made at the discretion of the clinical team. As an example, 5 patients were treated with thrombectomy and included in the analysis who were above 85 years old. Collateral profiles were defined using Tan and CBVI, though alternate methods may have produced different results. Tan was calculated by consensus between a board-certified neuroradiologist and vascular neurologist, rather than with independent reads and reporting of inter-rater reliability. It was also used for ICA occlusion patients rather than only MCA [23]. However, consensus Tan scoring has been previously reported, as has calculation for patients presenting with an ICA occlusion [33]. In addition, single-phase CTA scoring, rather than multi-phase, may have limited the ability of Tan to predict clinical outcomes [33].

Further, a larger sample size would have permitted greater power to detect differences in the collateral scoring groups and provided the opportunity to specifically evaluate more CCC+ patients. A larger sample would have also allowed additional adjustment of covariates in the regression model, such as treatment with IV thrombolysis. The specific combination of Tan < 2 and CBVI ≥ 0.8 was observed in only 1 patient, further emphasizing the limitations of a small sample size and limiting this specific comparison group. Missing values for baseline characteristics were imputed using the population median, which may attenuate the association between variables. Given the modest missingness, however, we judge this impact to be limited. Future studies with multiple imputations for subjects missing 90-day outcomes would be informative. CTP was performed with a single software program, though this has been the gold standard for use in numerous randomized trials [34,35,36]. Core overestimation is possible, particularly in early time windows, though the standard definition of CTP core size was used, as in prior randomized clinical thrombectomy studies [34,35,36]. While median core volume in our study was >70 mL, median ASPECTS was 8, highlighting the challenges of quantifying core size using different imaging modalities. Post-thrombectomy mTICI scores were obtained from the treating neuro-interventionalist and are thus subject to bias, though this has previously been reported to not affect the association between mTICI scores and functional outcomes [37]. Although the proportion of diabetic patients did not differ among collateral scoring groups, stroke outcomes have been reported to be worse overall in diabetic women compared to men. While not investigated here, future studies may further explore the interaction of sex and diabetes with outcomes after large core thrombectomy [38].

## 5. Conclusions

In summary, most patients in our large core thrombectomy cohort had poor collateral profiles as measured by single-phase CTA and CTP CBVI. No independent association was detected between favorable or mixed cerebral collateral circulation and 90-day mRS 0–3, though the study was underpowered for the CCC+ comparison. Presenting glucose was the only factor significantly associated with outcomes in our study. Additional trials with higher power may further explore the role of collateralization on infarct growth rate, penumbral volumes, and large core thrombectomy functional outcomes.

## Figures and Tables

**Table 1:** Baseline patient characteristics.

	Total N = 88
Demographics	
Age, years, median (IQR)	65.5 (56.5–77.5)
Sex, male, n (%)	48 (54.5%)
Race, n (%)	
Black	30 (34.1%)
White	53 (60.2%)
Other/unknown	5 (5.7%)
Medical history	
Pre-morbid mRS, median (IQR)	0 (0–1)
Hypertension, n (%)	56 (63.6%)
Hyperlipidemia, n (%)	40 (45.5%)
Diabetes mellitus, n (%)	24 (27.3%)
Coronary artery disease, n (%)	19 (21.6%)
Atrial Fibrillation, n (%)	22 (25.0%)
Smoking, n (%)	33 (37.5%)
Presentation Details	
Initial NIHSS, median (IQR)	19.0 (15.0–23.0)
Glucose (mg/dL), mean ± SD	143.1 (51.8)
Site of Occlusion, n (%)	
Internal Carotid Artery	35 (39.8%)
Middle Cerebral Artery—M1	41 (46.6%)
Middle Cerebral Artery—M2	12 (13.6%)
CT ASPECTS, median (IQR)	8.0 (5.0–10.0)
CBF < 30%, mL, median (IQR)	79.5 (57.0–117.0)
Tmax > 6 s, mL, median (IQR)	182.5 (144.0–256.0)
IV thrombolysis + thrombectomy, n (%)	20 (22.7%)
Thrombectomy only, n (%)	68 (77.3%)
TLKW to skin puncture (min), median (IQR)	325.0 (167.0–738.0)
mTICI 2b-3 revascularization, n (%)	77 (87.5%)
Collateral Assessments, CTP Parameters, and sICH	
Tan Score, median (IQR)	1.0 (1.0–2.0)
CBVI, median (IQR)	0.6 (0.5–0.7)
HIR, median (IQR)	0.7 (0.5–0.7)
Symptomatic intracranial hemorrhage, n (%)	2 (2.2%)

N, total number; IQR, interquartile range; n, number; mRS, modified Rankin Scale; NIHSS, National Institutes of Health Stroke Scale; mg, milligrams; dL, deciliter; SD, standard deviation; MCA, middle cerebral artery; CT, computed tomography; ASPECTS, Alberta Stroke Program Early Computed Tomography Score; CBF, cerebral blood flow; mL, milliliters; Tmax, time-to-maximum; IV, intravenous; TLKW, time last known well; min, minutes; mTICI, modified treatment in cerebral ischemia; CTP, computed tomography perfusion; sICH, symptomatic intracranial hemorrhage; CBVI, cerebral blood volume index; HIR, hypoperfusion intensity ratio.

**Table 2:** Patient characteristics stratified by collateral status.

	CCC− N = 47	CCC± N = 31	CCC+ N = 10	Total N = 88	*p*-Value
Demographics					
Age, years, median (IQR)	66.0 (58.0–76.0)	64.0 (55.0–78.0)	67.0 (49.0–79.0)	65.5 (56.5–77.5)	0.99
Sex, male, n (%)	31 (66.0%)	14 (45.2%)	3 (30.0%)	48 (54.5%)	0.051
Race, n (%)					0.95
Black	16 (34.0%)	11 (35.5%)	3 (30.0%)	30 (34.1%)	
White	27 (57.4%)	19 (61.3%)	7 (70.0%)	53 (60.2%)	
Other/unknown	4 (8.5%)	1 (3.2%)	0 (0.0%)	5 (5.7%)	
Medical history					
Pre-morbid mRS, median (IQR)	0 (0–1)	0 (0–2)	0 (0–0)	0 (0–1)	0.51
Hypertension, n (%)	30 (63.8%)	20 (64.5%)	6 (60.0%)	56 (63.6%)	1.00
Hyperlipidemia, n (%)	24 (51.1%)	14 (45.2%)	2 (20.0%)	40 (45.5%)	0.21
Diabetes mellitus, n (%)	17 (36.2%)	5 (16.1%)	2 (20.0%)	24 (27.3%)	0.14
Coronary artery disease, n (%)	12 (25.5%)	5 (16.1%)	2 (20.0%)	19 (21.6%)	0.62
Atrial Fibrillation, n (%)	11 (23.4%)	8 (25.8%)	3 (30.0%)	22 (25.0%)	0.88
Smoking, n (%)	14 (29.8%)	17 (54.8%)	2 (20.0%)	33 (37.5%)	0.04
Presentation Details					
Initial NIHSS, median (IQR)	19.0 (15.0–24.0)	19.0 (14.0–22.0)	17.0 (13.0–18.0)	19.0 (15.0–23.0)	0.09
Glucose (mg/dL), mean ± SD	143.2 (52.7)	145.0 (53.7)	137.0 (46.2)	143.1 (51.8)	0.92
Site of Occlusion, n (%)					0.018
Internal Carotid Artery	22 (46.8%)	7 (22.6%)	6 (60.0%)	35 (39.8%)	
Middle Cerebral Artery—M1	22 (46.8%)	15 (48.4%)	4 (40.0%)	41 (46.6%)	
Middle Cerebral Artery—M2	3 (6.4%)	9 (29.0%)	0 (0.0%)	12 (13.6%)	
CT ASPECTS, median (IQR)	9.0 (6.0–10.0)	7.0 (5.0–10.0)	5.5 (5.0–8.0)	8.0 (5.0–10.0)	0.037
CBF < 30% mL, median (IQR)	92.0 (65.0–153.0)	66.0 (52.0–85.0)	50.5 (36.0–83.0)	79.5 (57.0–117.0)	<0.001
IV thrombolysis, n (%)					0.29
IV thrombolysis + EVT	9 (19.1%)	10 (32.3%)	1 (10.0%)	20 (22.7%)	
EVT only	38 (80.9%)	21 (67.7%)	9 (90.0%)	68 (77.3%)	
TLKW to skin puncture (min), median (IQR)	250.5 (161.0–598.0)	326.0 (171.0–763.0)	651.5 (267.0–985.0)	325.0 (167.0–738.0)	0.14
Collateral Measures					
CTP CBVI, median (IQR)	0.5 (0.4–0.6)	0.6 (0.5–0.7)	0.8 (0.8–0.8)	0.6 (0.5–0.7)	<0.001
Tan score, median (IQR)	1.0 (1.0–1.0)	2.0 (2.0–2.0)	2.0 (2.0–3.0)	1.0 (1.0–2.0)	<0.001
Functional Outcomes					
90-day mRS score, median (IQR)	5.0 (3.0–6.0)	5.0 (3.0–6.0)	4.5 (3.0–6.0)	5.0 (3.0–6.0)	0.76
90-day mRS score 0–3, n (%)	15 (31.9%)	9 (29.0%)	4 (40.0%)	28 (31.8%)	0.85
90-day mRS score 0–2, n (%)	10 (21.3%)	5 (16.1%)	2 (20.0%)	17 (19.3%)	0.93
90-day mortality, n (%)	18 (38.3%)	15 (48.4%)	4 (40.0%)	37 (42.0%)	0.67

CCC, cerebral collateral cascade; N, total number; IQR, interquartile range; n, number; mRS, modified Rankin Scale; NIHSS, National Institutes of Health Stroke Scale; mg, milligrams; dL, deciliter; SD, standard deviation; MCA, middle cerebral artery; ACA, anterior cerebral artery; CT, computed tomography; ASPECTS, Alberta Stroke Program Early Computed Tomography Score; IV, intravenous; EVT, endovascular thrombectomy; TLKW, time last known well; min, minutes; CTP, computed tomography perfusion; CBVI, cerebral blood volume index.

**Table 3:** Multivariable Firth’s logistic regression for 90-day functional outcome (mRS 0–3 versus 4–6).

	Odds Ratio	*p*-Value	95% Confidence Interval	
Collateral status (ref: CCC−)				
CCC+	1.117	0.889	0.235	5.311
CCC±	0.869	0.800	0.293	2.577
Age	0.966	0.058	0.931	1.001
NIHSS	0.965	0.497	0.870	1.070
Glucose 10 mg/dL	0.742	0.005	0.602	0.915

mRS, modified Rankin Scale; CCC, cerebral collateral cascade; NIHSS, National Institutes of Health Stroke Scale; mg, milligrams; dL, deciliter.

## Data Availability

The raw data supporting the conclusions of this article will be made available by the authors on request.

## Supplementary Materials

The following supporting information can be downloaded at: https://www.mdpi.com/article/10.3390/jcm15218730/s1, Table S1: Distribution of Tan score and CBVI; Table S2: Missing Variable Distributions; Table S3: Results of Firth’s logistic regression with alternative CBVI thresholds.

## Abbreviations

The following abbreviations are used in this manuscript:

LVO	Large vessel occlusion
CTA	Computed tomography angiography
CTP	Computed tomography perfusion
EVT	Endovascular thrombectomy
mL	milliliters
CBVI	Cerebral blood volume index
DSA	Digital subtraction angiography
IRB	Institutional review board
ICA	Internal carotid artery
MCA	Middle cerebral artery
ASPECTS	Alberta Stroke Program Early Computed Tomography Score
CBF	Cerebral blood flow
mRS	Modified Rankin Scale
mTICI	Modified treatment in cerebral ischemia
NIHSS	National Institutes of Health Stroke Scale
CCC	Cerebral collateral cascade
IQR	Interquartile range
SD	Standard deviation
TLKW	Time last known well
HIR	Hypoperfusion intensity ratio
OR	Odds ratio
CI	Confidence interval
COVES	Cortical vein opacification score
AI	Artificial intelligence

## References

[1] Uniken Venema S.M., Dankbaar J.W., van der Lugt A., Dippel D.W.J., van der Worp H.B. (2022). Cerebral collateral circulation in the era of reperfusion therapies for acute ischemic stroke. Stroke.

[2] Maguida G., Shuaib A. (2023). Collateral circulation in ischemic stroke: An updated review. J. Stroke.

[3] Román L.S., Menon B.K., Blasco J., Hernández-Pérez M., Dávalos A., Majoie C.B.L.M., Campbell B.C.V., Guillemin F., Lingsma H., Anxionnat R. (2018). Imaging features and safety and efficacy of endovascular stroke treatment: A meta-analysis of individual patient-level data. Lancet Neurol..

[4] Menon B.K., O’Brien B., Bivard A., Spratt N.J., Demchuk A.M., Miteff F., Lu X., Levi C., Parsons M.W. (2013). Assessment of leptomeningeal collaterals using dynamic CT angiography in patients with acute ischemic stroke. J. Cereb. Blood Flow. Metab..

[5] Jansen I.G.H., van Vuuren A.B., van Zwam W.V., van den Wijngaard I.R., Berkhemer O., Lingsma H.F., Slump C.H., van Oostenbrugge R.J., Treurniet K.M., Dippel D.W.J. (2017). Absence of cortical vein opacification is associated with lack of intra-arterial therapy benefit in stroke. Radiology.

[6] Kakadiya J., Lakhani D.A., Salim H.A., Koneru M., Wolman D., Majmundar S., Hui F., Lu H., Dmytriw A., Guenego A. (2026). Prolonged venous transit on perfusion imaging: An evolving marker of outcomes in large vessel occlusion stroke—A comprehensive review. AJNR Am. J. Neuroradiol..

[7] Karamchandani R.R., Strong D., Rhoten J.B., Prasad T., Selig J., Defilipp G., Asimos A.W. (2022). Cerebral blood volume index as a predictor of functional independence after basilar artery thrombectomy. J. Neuroimaging.

[8] Karamchandani R.R., Yang H., Strong D., Rhoten J.B., Clemente J.D., Defilipp G., Patel N.M., Bernard J.D., Stetler W.R., Parish J.M. (2024). Glucose and cerebral blood volume index as predictors of ambulatory function for patients presenting with ultra-large core infarctions. Clin. Neuroimaging.

[9] Karamchandani R.R., Yang H., Teli K.J., Strong D., Rhoten J.B., Clemente J.D., Defilipp G., Patel N.M., Bernard J.D., Stetler W.R. (2025). Long-term functional outcomes for elderly patients treated with endovascular thrombectomy. Interv. Neuroradiol..

[10] Asimos A.W., Yang H., Strong D., Teli K.J., Clemente J.D., DeFilipp G., Bernard J., Stetler W., Parish J.M., Hines A. (2025). Association of hypoperfusion intensity ratio and cerebral blood volume Index with good outcome in patients transferred for thrombectomy. Interv. Neuroradiol..

[11] Sun A., Cao Y., Jia Z., Zhao L., Shi H., Liu S. (2023). Prognostic value of CBV index in patients with acute ischemic stroke treated with endovascular thrombectomy in late therapeutic window. Front. Neurol..

[12] Wan Z., Meng Z., Xie S., Fang J., Li L., Chen Z., Liu J., Jiang G. (2022). Correlation between Hypoperfusion Intensity Ratio and functional outcome in large-vessel occlusion acute ischemic stroke: Comparison with multi-phase CT angiography. J. Clin. Med..

[13] Faizy T.D., Mlynash M., Kabiri R., Christensen S., Kuraitis G.M., Mader M.M., Flottman F., Broocks G., Lansberg M.G., Albers G.W. (2022). The cerebral collateral cascade: Comprehensive blood flow in ischemic stroke. Neurology.

[14] Chen H., Colasurdo M. (2024). Endovascular thrombectomy for large ischemic strokes: Meta-analysis of six multicenter randomized controlled trials. J. Neurointerv. Surg..

[15] Gallardo A., Lavados P.M., Albiña-Palmarola P., Cavada G., Roldán A., Olavarría V.V. (2026). Collateral status predicts functional outcome in early-treated large-core anterior circulation stroke. Front. Stroke.

[16] Lai H., Huang J., Fan S., Liu K., Guo C., Tian Z., Deng W., Yang J., Li L., Song J. (2026). Prognostic value of collateral assessment in acute large-core infarction after endovascular thrombectomy. Acad. Radiol..

[17] Sarraj A., Hassan A.E., Abraham M.G., Chen M., Hussain M.S., Kasner S.E., Ortega-Gutierrez S., Churilov L., Pujara D., Shaker F. (2024). Abstract 40: Relationship of collateral status with clinical outcomes in endovascular thrombectomy for large core stroke: A SELECT2 subanalysis. Stroke.

[18] Winkelmeier L., Kniep H., Thomalla G., Bendszus M., Subtil F., Bonekamp S., Aamodt A.H., Fuentes B., Gizewski E.R., Hill M.D. (2025). Arterial collaterals and endovascular treatment effect in acute ischemic stroke with large infarct: A secondary analysis of the TENSION trial. Radiology.

[19] Sarraj A., Thomalla G., Yoshimura S., Huo X., Costalat V., Zaidat O.O., Arquizan C., Johns H., Yogendrakumar V., Churilov L. (2026). Endovascular thrombectomy for patients with large-core ischaemic stroke presenting up to 24 h after onset (ATLAS): A systematic review and individual patient data meta-analysis with central imaging adjudication. Lancet.

[20] Jovin T.G., Nogueira R.G., Lansberg M.G., Demchuk A.M., Martins S.O., Mocco J., Ribo M., Jadhav A.P., Ortega-Gutierrez S., Hill M.D. (2022). Thrombectomy for anterior circulation stroke beyond 6 h from time last known well (AURORA): A systematic review and individual patient data meta-analysis. Lancet.

[21] Goyal M., Menon B.K., van Zwam W.H., Dippel D.W., Mitchell P.J., Demchuk A.M., Dávalos A., Majoie C.B.L.M., va der Lugt A., de Miquel M.A. (2016). HERMES collaborators. Endovascular thrombectomy after large-vessel ischaemic stroke: A meta-analysis of individual patient data from five randomised trials. Lancet.

[22] Almekhlafi M.A., Mishra S., Desai J.A., Nambiar V., Volny O., Goel A., Eesa M., Demchuk A., Menon B.K., Goyal M. (2014). Not all “successful” angiographic reperfusion patients are an equal validation of a modified TICI scoring system. Interv. Neuroradiol..

[23] Tan I.Y.L., Demchuk A.M., Hopyan J., Zhang L., Gladstone D., Wong K., Martin M., Symons S.P., Fox A.J., Aviv R.I. (2009). CT angiography clot burden score and collateral score: Correlation with clinical and radiologic outcomes in acute middle cerebral artery infarct. AJNR Am. J. Neuroradiol..

[24] Molaie A., Saver J.L. (2026). A key distinction in EVT decision-making: Accurately measured core volume is a prognostic marker and accurately measured penumbra volume is a predictive biomarker. Stroke Vasc. Interv. Neurol..

[25] Ter Schiphorst A., Seners P., Arquizan C., Yedavalli V., Olivot J.-M., Lansberg M.G., Muir K.W., Parsons M.W., Saver J.L., Fisher M. (2025). The large core paradox. Stroke.

[26] Yogendrakumar V., Campbell B.C.V., Johns H., Churilov L., Ng F.C., Sitton C.W., Hassan A.E., Abraham M.G., Ortega-Gutierrez S., Hussain M.S. (2025). Association of ischemic core hypodensity with thrombectomy treatment effect in large core stroke: A secondary analysis of the SELECT2 Randomized Controlled Trial. Stroke.

[27] Saposnik G., Kapral M.K., Liu Y., Hall R., O’Donnell M., Raptis S., Tu J.V., Mamdani M., Austin P.C. (2011). Investigators of the Registry of the Canadien Stroke Network; Stroke Outcomes Research Canda (SORCan) Working Group. IScore: A risk score to predict death early after hospitalization for an acute ischemic stroke. Circulation.

[28] Hallevi H., Barreto A.D., Liebeskind D.S., Morales M.M., Martin-Schild S.B., Abraham A.T., Gadia J., Saver J.L., Grotta J.C., UCALAIntra-Arterial Therapy Investigators (2009). Identifying patients at high risk for poor outcome after intra-arterial therapy for acute ischemic stroke. Stroke.

[29] Sarraj A., Albright K., Barreto A.D., Boehme A.K., Sitton C.W., Choi J., Lutzker S.L., Sun C.H.J., Bibars W., Nguyen C.B. (2013). Optimizing prediction scores for poor outcome after intra-arterial therapy in anterior circulation acute ischemic stroke. Stroke.

[30] Karamchandani R.R., Prasad T., Strong D., Rhoten J.B., Asimos A.W. (2022). A tool to improve stroke outcome prediction: The charlotte large artery occlusion endovascular therapy outcome score. J. Stroke Cerebrovasc. Dis..

[31] Ferrari F., Moretti A., Villa R.F. (2022). Hyperglycemia in acute ischemic stroke: Physiopathological and therapeutic complexity. Neural Regen. Res..

[32] Johnston K.C., Bruno A., Pauls Q., Hall C.E., Barrett K.M., Barsan W., Fansler A., Van de Bruinhorst K., Janis S., Dukalski-Mauldin V. (2019). Neurological Emergencies Treatment Trials Network and the SHINE Trial Investigators. Intensive vs Standard Treatment of Hyperglycemia and Functional Outcome in Patients with Acute Ischemic Stroke: The SHINE Randomized Clinical Trial. JAMA.

[33] Gensicke H., Al-Ajlan F., Fladt J., Campbell B.C.V., Majoie C.B.L.M., Bracard S., Hill M.D., Muir K.W., Demchuk A., San Román L. (2022). Comparison of three scores of collateral status for their association with clinical outcome: The HERMES collaboration. Stroke.

[34] Albers G.W., Marks M.P., Kemp S., Christensen S., Tsai J.P., Ortega-Gutierrez S., McTaggart R.A., Torbey M.T., Kim-Tenser M., Leslie-Mazwi T. (2018). Thrombectomy for stroke at 6 to 16 hours with selection by perfusion imaging. N. Engl. J. Med..

[35] Nogueira R.G., Jadhav A.P., Haussen D.C., Bonafe A., Budzik R.F., Bhuva P., Yavagal D.R., Ribo M., Cognard C., Hanel R.A. (2018). Thrombectomy 6 to 24 hours after stroke with a mismatch between deficit and infarct. N. Engl. J. Med..

[36] Sarraj A., Hassan A.E., Abraham M.G., Ortega-Gutierrez S., Kasner S.E., Hussain M.S., Chen M., Blackburn S., Sitton C.W., Churilov L. (2023). Trial of Endovascular Thrombectomy for Large Ischemic Strokes. N. Engl. J. Med..

[37] Zhang G., Treurniet K.M., Jansen I.G.H., Emmer B.J., van den Berg R., Marquering H.A., Uyttenboogaart M., Jenniskens S.F.M., Roos Y.B.W.E.M., van Doormaal P.J. (2018). Operator versus core lab adjudication of reperfusion after Endovascular Treatment of Acute Ischemic Stroke. Stroke.

[38] Peters S.A.E., Huxley R.R., Woodward M. (2014). Diabetes as a risk factor for stroke in women compared with men: A systematic review and meta-analysis of 64 cohorts, including 775,385 individuals and 12,539 strokes. Lancet.

