# Supplementary material for: Association of Multimodal Collateral Profiles with Outcomes After Large Core Thrombectomy
Source: J Clin Med. 2026 Nov 9;15(21):8730. doi: 10.3390/jcm15218730 (PMC13764456; doi:10.3390/jcm15218730)
Supplement: Supplementary file 1 [file jcm-15-08730-s001.zip › jcm-4656230-supplementary.pdf]

<u>**Supplemental Material**</u>

Supplemental Table S1. Distribution of Tan score and CBVI

	CBVI <0.8	CBVI ≥0.8	CBVI missing	Row total
Tan score <2	49 (53.85%)	1 (1.10%)	3 (3.30%)	53 (58.24%)
Tan score ≥2	24 (26.37%)	10 (10.99%)	3 (3.30%)	37 (40.66%)
Tan score missing	1 (1.10%)	0 (0%)	0 (0%)	1 (1.10%)
Column total	74 (81.32%)	11 (12.09%)	6 (6.59%)	91 (100%)

Supplemental Table S2. Missing Variable Distributions

Variable	N missing	% missing	Imputation method
Tan score	1 / 91	1.1%	Population median
CBVI	6 / 91	6.6%	Population median
Admission glucose	6 / 91	6.6%	Population median
90-day mRS (primary outcome)	3 / 91	3.3%	Population median

Variable selection and model evaluation

Due to the limited number of events relative to candidate predictors (EPV constraint), covariates were selected based on established clinical relevance rather than statistical significance within this cohort. Age, NIHSS, and glucose were included as consistent independent predictors of 90-day functional outcome. We did not include mTICI 2b-3 in the model as it is assessed after the exposure of interest and is thus considered a mediator rather than a confounder. Occlusion site differs across collateral groups (p = 0.030) but was not independently associated with the primary outcome (p = 0.300) and was therefore not retained in the final model. IV thrombolysis was balanced across collateral groups (p = 0.238) and was not independently associated with outcome (p =

0.371) so was also excluded. We did not include other imaging or clinical covariates, such as HIR, because of the EPV constraint.

We fitted the primary multivariable model using Firth's penalized logistic regression, with the covariate set, including collateral status (CCC), age, baseline NIHSS, and admission glucose. The EPV was 5.6. The apparent C-statistic was 0.799; the mean bootstrap-estimated optimism was 0.044, resulting in an optimism-corrected C-statistic of 0.755. The optimism-corrected estimate continues to indicate reasonable discriminative ability, and the magnitude of optimism is not suggestive of severe overfitting. For the calibration, the mean predicted probability is 0.326 and the observed event rate is 0.318. These results show no evidence of systematic over or under prediction. Our Firth's penalized logistic regression was developed to estimate an adjusted association for a prespecified exposure of interest rather than to serve as a clinical prediction tool. These metrics are reported as supplementary model-fit information.

Sensitivity Analysis

As a robustness check, we repeated the primary analysis after imputing three missing 90-day mRS patients with the median imputation method (N = 91). Results were consistent between the two approaches, including the primary finding for glucose (OR 0.752, 95% CI 0.615-0.918, p = 0.005 in the N = 91 analysis vs. OR 0.742, 95% CI 0.602-0.915, p = 0.005, N = 88)

Supplementary Table S3. Results of Firth's logistic regression with alternative CBVI thresholds

CBVI threshold	Glucose OR (per 10 mg/dL)
0.7	0.736, p=0.004
0.8 (primary)	0.742, p=0.005
0.9	0.743, p=0.005

The association between admission glucose and functional outcome remained stable and significant across all 3 CBVI thresholds.
